# Supplementary material for: A Comparative Study of Morphology, Photosynthetic Physiology, and Proteome between Diploid and Tetraploid Watermelon (Citrullus lanatus L.)
Source: Bioengineering (Basel). 2022 Dec 1;9(12):746. doi: 10.3390/bioengineering9120746 (PMC9774721; doi:10.3390/bioengineering9120746)
Supplement: Supplementary file 1 [file bioengineering-09-00746-s001.zip › bioengineering-2028509-supplementary.pdf]

Table S1 Primers sequence used for qRT-PCR

| Gene ID | Forward Primer | Reverse Primer |
|---|---|---|
| Cla009752 | CGTGAGCTCGAAGTGATCCA | CGTGAGCTCGAAGTGATCCA |
| Cla010223 | CTGTCTTGGCCACTACACGT | TCGAACCGTGGTCCTCAAAG |
| Cla011786 | TGGCTACGGTTCTCGATTCC | AGCGATCTTCAAGCCTCTGC |
| Cla007717 | GGTCAGAGGACCTGCACCTT | ACCATGGATTCTCGCCAGTT |
| Cla008848 | AGAGAGGCCACAGCCTTGAG | CGTCGGCAACACTTCCACTA |
| Cla007792 | CCATGTATGTTGCCATCCAG | GGATAGCATGGGGTAGAGCA |
